# Dosage-Dependent Impact of Acute Serotonin Enhancement on Transcranial Direct Current Stimulation Effects

**DOI:** 10.1093/ijnp/pyab035

**Published:** 2021-06-09

**Authors:** Lorena Melo, Mohsen Mosayebi-Samani, Elham Ghanavati, Michael A Nitsche, Min-Fang Kuo

**Affiliations:** 1 Department of Psychology and Neurosciences, Leibniz Research Centre for Working Environment and Human Factors (IfADo), Dortmund, Germany; 2 International Graduate School of Neuroscience (IGSN), Ruhr-University Bochum, Germany; 3 Department of Neurology, University Medical Hospital Bergmannsheil, Bochum, Germany

**Keywords:** Serotonin, transcranial direct current stimulation, motor cortex, neuroplasticity

## Abstract

**Background:**

The serotonergic system has an important impact on basic physiological and higher brain functions. Acute and chronic enhancement of serotonin levels via selective serotonin reuptake inhibitor administration impacts neuroplasticity in humans, as shown by its effects on cortical excitability alterations induced by non-invasive brain stimulation, including transcranial direct current stimulation (tDCS). Nevertheless, the interaction between serotonin activation and neuroplasticity is not fully understood, particularly considering dose-dependent effects. Our goal was to explore dosage-dependent effects of acute serotonin enhancement on stimulation-induced plasticity in healthy individuals.

**Methods:**

Twelve healthy adults participated in 7 sessions conducted in a crossover, partially double-blinded, randomized, and sham-controlled study design. Anodal and cathodal tDCS was applied to the motor cortex under selective serotonin reuptake inhibitor (20 mg/40 mg citalopram) or placebo medication. Motor cortex excitability was monitored by single-pulse transcranial magnetic stimulation.

**Results:**

Under placebo medication, anodal tDCS enhanced, and cathodal tDCS reduced, excitability for approximately 60–120 minutes after the intervention. Citalopram enhanced and prolonged the facilitation induced by anodal tDCS regardless of the dosage while turning cathodal tDCS-induced excitability diminution into facilitation. For the latter, prolonged effects were observed when 40 mg was administrated.

**Conclusions:**

Acute serotonin enhancement modulates tDCS after-effects and has largely similar modulatory effects on motor cortex neuroplasticity regardless of the specific dosage. A minor dosage-dependent effect was observed only for cathodal tDCS. The present findings support the concept of boosting the neuroplastic effects of anodal tDCS by serotonergic enhancement, a potential clinical approach for the treatment of neurological and psychiatric disorders.

**Significance Statement**

This study shows that in healthy humans, acute serotonin enhancement on the motor cortex influences neuroplasticity, the basis for the adaptive capacity of the brain, which is reduced in depression and relevant for brain recovery after injury. Citalopram enhanced neuroplasticity induced by anodal transcranial direct current stimulation (tDCS), a non-invasive brain stimulation tool, largely independently from the administered dosage (20 or 40 mg). Our results provide further insights into boosting the effects of anodal tDCS, which is probed as an antidepressant agent, and for the treatment of other brain diseases by serotonergic enhancement.

## Introduction

Due to its extensive innervation of cortical and subcortical regions, the serotonergic system has a remarkable role from basic physiological to higher brain functions (Mann, 1999; Ciranna, 2006). Studies in humans and animals have revealed a relevant impact of serotonin (5-hydroxytryptamine [5-HT]) on neuroplasticity (Bert et al., 2008; Ogren et al., 2008; Kraus et al., 2017), the basis for the adaptive capacity of the central nervous system (Kleim and Jones, 2008). Neuroplasticity mechanisms are related to the efficacy of synaptic connections. Synapses can strengthen (via long-term potentiation [LTP]) or weaken (via long-term depression [LTD]) their efficacy, driven by a stimulus-dependent increase or decrease in their activity. Animal studies showed that 5-HT modulates LTP and LTD, and these effects are associated with receptor subtypes, dosage, and duration of 5-HT receptor activation (Kojic et al., 1997; Kemp and Manahan-Vaughan, 2005). However, knowledge about serotonergic modulation of neuroplasticity in humans is limited.

The effect of selective 5-HT reuptake inhibitors (SSRI) on human brain physiology has been investigated via plasticity-inducing non-invasive brain stimulation (NIBS) approaches, including paired associative stimulation (PAS) (Batsikadze et al., 2013a), and transcranial direct current stimulation (tDCS) (Nitsche et al., 2009; Kuo et al., 2016). In the past 20 years, the mechanistic aspects of tDCS have been largely explored for the application in clinical scenarios. tDCS induces neuroplasticity non-invasively through the application of low-intensity current to the brain (Woods et al., 2016). For standard protocols, albeit extensively investigated for the motor cortex, tDCS effects are polarity dependent. As a primary effect, anodal tDCS induces a neuronal membrane depolarization via subthreshold electrical current, whereas cathodal tDCS induces the opposite effect by neuronal membrane hyperpolarization (Nitsche and Paulus, 2000, 2001). At the macroscopic level, this translates, respectively, into enhancement or reduction of cortical excitability (Lefaucheur et al., 2017). Additionally, sufficient stimulation duration results in calcium-dependent glutamatergic plasticity induction as a secondary effect demonstrated by pharmacological studies (Nitsche et al., 2003b, 2004; Martins et al., 2019; Mosayebi-Samani et al., 2020). Animal studies indicate that moderate and prolonged intracellular calcium influx causes LTD, while a larger calcium influx results in LTP (Lisman, 2001; Malenka and Bear, 2004). Likewise, LTD can be switched to LTP via intensified intervention protocols. Moreover, calcium overflow by further intensifying interventions has been shown to result in counterregulatory mechanisms preventing LTP (Cummings et al., 1996; Lisman, 2001).

Similarly, tDCS after-effects are influenced by calcium dynamics (Nitsche et al., 2003b; Mosayebi-Samani et al., 2020) are dependent on stimulation parameters (Monte-Silva et al., 2013) and, can also be non-linear (Batsikadze et al., 2013b; Mosayebi Samani et al., 2019; Hassanzahraee et al., 2020). These effects likely rely on combined polarization and alteration of spontaneous activity (Fritsch et al., 2010), which is well aligned with general plasticity mechanisms in animal models. How tDCS specifically interacts with ongoing synaptic activity to induce plasticity is still under investigation. Nevertheless, anodal and cathodal tDCS are assumed to induce LTP- and LTD-like effects (Nitsche et al., 2003b; Kronberg et al., 2017) in humans, and the after-effects are associated with N-methyl-d-aspartate receptor (NMDAR) and calcium-dependent plasticity of the glutamatergic system (Liebetanz et al., 2002). Changes in γ-aminobutyric acid (GABA) activity, which is reduced by both cathodal and anodal tDCS (Stagg et al., 2009), are assumed to have a “gating role” to trigger glutamatergic plasticity, such that the interaction of GABA and glutamate alterations determine the propensity of tDCS-induced plasticity (Heimrath et al., 2020).

Due to possible synergistic effects, there is growing interest to investigate the association between plasticity-inducing techniques and pharmacological approaches. It has been shown that the enhancement of 5-HT levels by SSRI modulates NIBS effects on the motor (Nitsche et al., 2009; Batsikadze et al., 2013a; Kuo et al., 2016) and temporoparietal cortex (Prehn et al., 2017), leading to enhanced facilitatory plasticity and improved memory formation in healthy participants. More specifically, a single application of 20 mg citalopram (CIT) prolonged LTP-like plasticity induced by anodal tDCS as well as excitatory PAS, whereas it converted the after-effects of cathodal tDCS into excitatory plasticity and reduced the inhibitory PAS effect (Nitsche et al., 2009; Batsikadze et al., 2013a). Chronic application of 20 mg CIT resulted in more extended LTP-like plasticity, lasting approximately 24 hours after anodal tDCS, compared with 4–5 hours for a single dose. Furthermore, these effects were abolished by dextromethorphan (an NMDAR antagonist), suggesting their dependence on NMDA receptor activity (Kuo et al., 2016) and stressing the modulatory role of 5-HT on glutamatergic plasticity. This synergistic effect of tDCS and SSRI has clinical relevance. The combination of 50 mg sertraline (an SSRI) and tDCS in patients with moderate to severe unipolar major depression showed a superior impact on major depression compared with placebo (PLC) and the respective single interventions (Brunoni et al., 2013).

Complex dosage-dependent effects of other neuromodulators on brain physiology and performance in humans have been demonstrated, such as for dopamine (Fresnoza et al., 2014; Chen et al., 2020). For 5-HT, a dose-dependent enhancement on motor dexterity and motor output (assessed via functional magnetic resonance imaging - fMRI) was found after administering 20 mg paroxetine (optimal dosage) compared with 60 mg (Loubinoux et al., 2002a). However, mechanistic knowledge about the dosage-dependent effect of serotonergic enhancement on plasticity in humans is not presently available. In this study, we aimed to investigate dosage-dependent effects of acute 5-HT enhancement on motor cortex plasticity in healthy humans. We hypothesized that 20 mg CIT increases and promotes LTP-like plasticity induced by anodal tDCS, while it converts the effects of cathodal tDCS from LTD- to LTP-like plasticity. Due to stronger calcium influx, for the higher dosage of CIT (40 mg), we hypothesized that respective effects might be strengthened or converted due to calcium overflow. The results will not only provide mechanistic knowledge but may also help fine-tune plasticity modulation in the human brain.

## Methods

### Participants

Twelve healthy individuals (6 females, 27.08 ± 6.58 years old) participated in the study. All participants were right-handed according to the Edinburgh Handedness Inventory (Oldfield, 1971), non-smokers, and were examined by a physician to assess health conditions as well as contraindications for NIBS and CIT intake. Volunteers with a present or prior neurological or psychiatric disease, history of epileptic seizures, substance addiction, regular intake of central nervous system-acting medication, current pregnancy, cardiac pacemaker, or metallic implants in the head were excluded. This study was conducted according to the Declaration of Helsinki and approved by the local ethics committee. All participants gave written informed consent before starting the study and were financially compensated.

### Monitoring Cortical Excitability

Motor-evoked potentials (MEPs) were obtained over the left primary motor cortex by single-pulse transcranial magnetic stimulation (TMS), with a figure-of-8 coil (diameter, 70 mm; peak magnetic field, 2 Tesla) connected to a PowerMag stimulator (Mag&More, Munich, Germany). Surface electromyography was recorded from the right abductor digiti minimi muscle (ADM) using Ag-AgCl electrodes in a belly-tendon montage. The optimal stimulation position (hotspot) was defined as the site where TMS pulses applied with medium intensity resulted consistently in the largest MEP of the right ADM. The coil was held tangentially to the skull, with the handle pointing backwards at an angle of 45° from the midline. The analog electromyography signals were amplified, band pass-filtered at 2 Hz–2 kHz, digitized at a sampling rate of 5 kHz (CED, Cambridge, UK), controlled by Signal Software (CED, v.2.13), and stored offline for further analyses.

### Pharmacological Intervention

CIT in dosages of 20 mg and 40 mg or equivalent PLC medication was administrated 2 hours before tDCS, immediately after determining the first TMS baseline excitability (baseline 1), to assure application of tDCS during peak plasma concentration (Bezchlibnyk-Butler et al., 2000). CIT inhibits the 5-HT transporter and enhances the availability of 5-HT in the somatodendritic area of serotonergic neurons and has no or very low affinity to other neurotransmitter systems (Stahl, 2013).

### tDCS

Low-intensity direct current was applied by a battery-driven DC stimulator (NeuroConn, Germany) through a pair of rubber electrodes embedded in 35-cm^2^ saline-soaked sponges. The target electrode was placed over the representation area of the right ADM with an angle orientation of 45° from the midsagittal line, and the return electrode was positioned above the contralateral supraorbital region (Foerster et al., 2019). The stimulation was applied with 1 mA for 13 minutes (anodal) or 9 minutes (cathodal) (Nitsche and Paulus, 2001; Nitsche et al., 2003a), with 15-second ramping-up and -down of stimulation intensity. For sham tDCS, the stimulation was applied for only 30 seconds; however, the electrodes remained on the head for 13 or 9 minutes (Nitsche et al., 2008).

### Experimental Procedures

**Figure 1.**
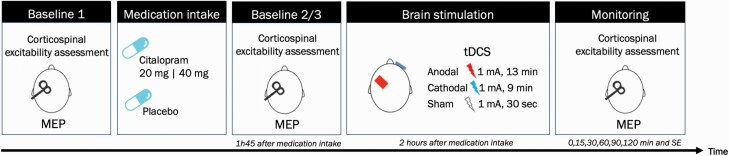
Experimental procedure. Schematic representation of the experimental procedure. In each session, participants were submitted to a combination of transcranial direct current stimulation (tDCS) and medication. Citalopram-dependent excitability alterations were monitored before tDCS (baseline 1, 2/3). tDCS after-effects were monitored via motor evoked potentials (MEPs) recordings immediately after tDCS, every 15 minutes until 30 minutes, every 30 minutes until 2 hours, and same-day evening (5–6 hours after stimulation).

The study was conducted in a crossover, randomized, sham-controlled, and partially double-blinded design. Participants were blinded for medication and tDCS conditions, and the experimenter was blinded for the medication. Participants were instructed not to consume alcohol for 24 hours and caffeine for at least 3 hours before the beginning of the experimental sessions. Session starting times were established by the chronotype of each participant, evaluated by the Morningness-Eveningness Questionnaire Self-Assessment Version. All participants underwent 7 sessions (Figure 1) with a washout period of at least 1 week between sessions to minimize carry-over effects.

In each session, participants sat in a comfortable reclining chair with head and armrests and were instructed to remain relaxed. An inflatable pillow was placed around their necks to stabilize the head position. Initially, the motor cortex hotspot was identified, and the position of the TMS coil and ADM electrodes was marked with a waterproof pen to guarantee stable positions throughout the session. TMS intensity was adjusted to elicit a baseline MEP average of 1 mV peak-to-peak amplitude (baseline 1). Immediately after obtaining baseline 1, participants received the pharmacological intervention (see above). A second baseline was recorded 1 hour and forty-five minutes after medication intake to monitor the influence of the substance on cortical excitability (baseline 2), and, when necessary, TMS intensity was adjusted to result in baseline MEP amplitudes of 1 mV (baseline 3). After determination of baseline 2/3, TMS intensity was kept constant for the post-tDCS measurements. Thirty MEPs were obtained for each cortical excitability assessment. Next, tDCS was applied 2 hours after medication intake. Immediately after tDCS, MEPs were recorded at the time points of 0, 15, 30, 60, 90, and 120 minutes and 5–6 hours after tDCS (same-day evening [SE]). At the end of each session, participants reported the presence or absence of adverse effects from tDCS (Brunoni et al., 2011), any additional side effects of medication, and if they thought to have received real or sham tDCS.

### Data Analysis and Statistics

The presence of adverse effects due to CIT intake and during and after tDCS was analyzed by 1-way repeated-measures ANOVAs considering medication (20 mg, 40 mg, and PLC) and stimulation protocols (anodal, cathodal, and sham), respectively, as within-subject factor. Blinding was evaluated using a chi-square test to assess whether participants could correctly guess the stimulation conditions. Pearson’s correlation was used to investigate an association between chronotype scores and MEP amplitudes (average of 0–30 minutes after stimulation; early epoch) for all conditions.

Individual peak-to-peak MEP amplitudes were first visually inspected to exclude trials in which muscle activity before the TMS pulse was present. MEP amplitude means were calculated for each time point and for each stimulation while medication conditions were separated per participant. For the main analysis, post-tDCS measurements were normalized to baseline 2 when TMS intensity had not been adjusted; otherwise, it was normalized to baseline 3. Because differences between baseline measurements may have an impact on primary outcome measures, differences between baseline MEP amplitudes and baseline TMS intensities (percentage of maximal stimulator output [%MSO]) were explored for non-normalized data. One-way ANOVAs considering “condition” (sham+PLC; cathodal+PLC; anodal+PLC; cathodal+CIT20mg; anodal+CIT20mg; cathodal+CIT40mg; anodal+CIT40mg) as within-subject factor were conducted for baselines 1 and 2/3. Then, to explore if CIT and PLC medications had an influence on cortical excitability before tDCS application, 2 additional 2-way repeated-measures ANOVA were calculated with condition and time (before and post-medication) as within-subject factors and MEP amplitudes or TMS intensities as dependent variables.

To identify if the intervention combinations had discernible effects on motor cortex excitability compared with the sham+PLC combination, a repeated-measures ANOVA was performed with condition (7 levels) and time-point (8 levels) as within-subject factors and normalized MEPs as the dependent variable. Moreover, to analyze the dose-dependent effect of CIT on MEP amplitudes over time, another repeated-measures ANOVA was calculated considering tDCS polarity (anodal and cathodal), substance (CIT 20 mg, CIT 40 mg, and PLC) and time-point (baseline and 0, 15, 30, 60, 90, 120 minutes, SE) as within-subject factors and normalized MEPs as dependent variable. An additional analysis to compensate for variability between single measurement time-points was conducted for post-stimulation effects pooled into 3 epochs: early (0–30 minutes), late (60–120 minutes), and very late (SE). A 2-way ANOVA was calculated with condition and time epochs as within-subject factors and normalized MEPs as dependent variable. Moreover, 3-way ANOVA was conducted considering tDCS polarity, substance, and time epochs as within-subject factors and normalized MEPs as dependent variable for the pooled data set.

Mauchly’s test of sphericity was conducted, and when necessary, the Greenhouse-Geisser correction was applied. When the respective ANOVA results showed significances, exploratory post-hoc Student’s *t* tests (paired samples, 2-tailed, *P* < .05, not corrected for multiple comparisons) were conducted to detect significant differences between baseline and tDCS after-effects, and between conditions. All statistical analyses were performed with SPSS version 26.0 (IBM SPSS Statistics, New York, NY, USA).

## Results

All participants tolerated tDCS well, and no significant difference of side effects was found between the stimulation protocols. The most common and frequently reported side effects were mild itching, tingling, burning sensation, and skin redness (supplementary Tables 1 and 2). Also, adverse effects of CIT did not significantly differ between dosages and were reported as light to moderate sleepiness, dizziness, fatigue, headache, nausea, and gastrointestinal discomfort, mainly when the 40-mg dosage was administrated (supplementary Tables 3 and 4). No participant dropped out due to adverse effects of tDCS or medication. Moreover, we found no significant difference in the chi-square test result (χ ^2^ = 4.015, df = 6, *P* = .675), suggesting that blinding was not compromised (supplementary Table 5). No significant correlation was found between chronotype scores and MEP amplitudes (sham+PLC = r(10) = −0.028, *P* = .535; cathodal+PLC = r(10) = 0.317, *P* = .842; anodal+PLC = r(10) = −0.06, *P* = .424; cathodal+20mg = r(10) = 0.346, *P* = .864); anodal+20mg = r(10) = −0.397, *P* = .10; cathodal+ 40mg = r(10) = −0.235, *P* = .231; anodal+40mg = r(10) = −0.418, *P* = .088), showing that we successfully controlled for possible chronotype- and daytime-dependent plasticity differences by establishing the optimal time to start the session for each participant.

### Comparison of Baseline MEP Amplitudes and %MSO Between Conditions

The repeated-measures 1-way ANOVA showed no significant differences of MEP amplitude values for baseline 1 (F_(6,66)_ = 0.571, η _p_^2^ = 0.049, *P* = .752) and baseline 2/3 (F_(6, 66)_ = 0.917, η _p_^2^ = 0.077, *P* = .489) across all conditions. The repeated-measures 2-way ANOVA revealed no significant main effects or interaction for the MEP amplitudes between baseline 1 and 2 (condition: F_(6,66)_ = 0.961, η _p_^2^ = 0.080, *P* = .458; time: F_(1,11)_ = 4.789, η _p_^2^ = 0.303, *P* = .051; condition × time: F_(6,66)_ = 0.622, η _p_^2^ = 0.053, *P* = .712), implying medication did not significantly affect cortical excitability. Likewise, the repeated-measures 1-way ANOVA showed no significant differences of %MSO for baseline 1 (F_[3.290,36.191]_ = 0.966, η _p_^2^ = 0.081, *P* = .455) and baseline 2/3 (F_(6,66)_ = 1.103, η _p_^2^ = 0.091, *P* = .370) across all conditions. The repeated-measures 2-way ANOVA showed no significant main effect for %MSO between baseline 1 and 3 (condition: F_(6,66)_ = 1.063, η _p_^2^ = 0.088, *P* = .394; time: F_(1,11)_ = 4.726, η _p_^2^ = 0.300, *P* = .052; condition × time: F_[3.539,38.929]_ = 0.461, η _p_^2^ = 0.040, *P* = .741). Baseline MEPs amplitudes and %MSO are listed in Table 1.

**Table 1.** MEP Amplitudes and Stimulation Intensity (%MSO) Before and After Substance Administration

		MEP Amplitude (mV)			%MSO	
tDCS	Medication	Baseline 1	Baseline 2	Baseline 3	Baseline 1	Baseline 3
Sham	Placebo	0.97 ± 0.05	0.97 ± 0.13	1.01 ± 0.08	53.25 ± 11.01	53.25 ± 11.01
Anodal	Placebo	1.01 ± 0.49	1.06 ± 0.19	1.00 ± 0.07	53.70 ± 7.44	53.58 ± 7.40
	CIT 20 mg	0.97 ± 0.06	1.02 ± 0.45	0.97 ± 0.06	53.33 ± 10.31	53.20 ± 10.83
	CIT 40 mg	1.00 ± 0.07	1.18 ± 0.36	1.01 ± 0.09	53.79 ± 8.51	53.04 ± 8.62
Cathodal	Placebo	1.00 ± 0.84	1.07 ± 0.38	0.97 ± 0.07	52.00 ± 9.65	51.45 ± 9.82
	CIT 20 mg	1.01 ± 0.09	1.12 ± 0.24	1.01 ± 0.04	52.33 ± 9.20	51.79 ± 9.15
	CIT 40 mg	0.98 ± 0.07	1.19 ± 0.38	1.01 ± 0.08	51.33 ± 10.33	51.04 ± 9.62

CIT = citalopram, MEP = motor evoked potential, %MSO = percentage of maximal stimulator output – the stimulation intensity, mV = milivolts, tDCS = transcranial direct current stimulation.

Baseline 1 refers to the MEP measured at the beginning of each session, baseline 2 refers to the MEP measured 1 hour and 45 minutes after medication intake, and baseline 3 refers to the MEP measurement immediately conducted after baseline 2 if transcranial magnetic stimulation intensity adjustment was necessary. Data are presented as mean ± standard deviation.

### Effect of CIT on tDCS-Induced Neuroplasticity (Overall Time Course)

The repeated-measures ANOVA conducted over all conditions, including sham+PLC, revealed significant main effects of condition (F_(6,66)_ = 9.671, η _p_^2^ = 0.468, *P* < .001) and time-point (F_(7,77)_ = 13.702, η _p_^2^ = 0.555, *P* < .001) and a significant condition × time-point interaction (F_(42,462)_ = 3.508, η _p_^2^ = 0.429, *P* < .001), indicating a difference between real intervention compared with the sham+PLC condition. Additionally, the repeated-measures ANOVA conducted to discern effects of stimulation and substance showed significant main effects of tDCS polarity (F_(1,11)_ = 8.273, η _p_^2^ = 0.468, *P* = .015) and time-point (F_(2,22)_ = 14.011, η _p_^2^ = 0.560, *P* < .001). Significant interactions were revealed for tDCS polarity × substance (F_(2,22)_ = 11.404, η _p_^2^ = 0.509, *P* < .001), tDCS polarity × time-point (F_(7,77)_ = 5.133, η _p_^2^ = 0.318, *P* < .001), and tDCS polarity × substance × time-point (F_(14,154)_ = 5.224, η _p_^2^ = 0.322, *P* < .001), revealing a discernible impact of dosage on tDCS conditions. However, the main effect of substance (F_(2,22)_ = 2.902, η _p_^2^ = 0.209, *P* = .076) and substance × time-point interactions (F_(14,154)_ = 1.073, η _p_^2^ = 0.089, *P* = .386) were not significant (Table 2).

**Table 2.** Results of Repeated-Measures ANOVAs for Overall and Pooled (Epochs) Data

	Factor	d.f., error	F value	η^2^_p_	*P* value
**Overall data analysis**	Condition	6, 66	9.671	0.468	<.001*
	Time-point	7, 77	13.702	0.555	<.001*
	Condition × time-point	42, 462	3.508	0.242	<.001*
	tDCS polarity	1, 11	8.273	0.429	.015*
	Substance	2, 22	2.902	0.209	.076
	Time-point	7, 77	14.011	0.560	<.001*
	tDCS polarity × substance	2, 22	11.404	0.509	<.001*
	tDCS polarity × time-point	7, 77	5.133	0.318	<.001*
	Substance × time-point	14, 154	1.073	0.089	.386
	tDCS polarity × substance × time-point	14, 154	5.224	0.322	<.001*
***Pooled data analysis***	Condition	6, 66	8.559	0.438	<.001*
	Epochs	2, 22	10.082	0.478	.001*
	Condition × epochs	12, 132	4.424	0.287	<.001*
	tDCS polarity	1, 11	8.876	0.447	.013*
	Substance	2, 22	2.193	0.166	.135
	Epochs	2, 22	10.810	0.496	.001*
	tDCS polarity × substance	2, 22	8.075	0.423	.002*
	tDCS polarity × epochs	1.350, 14.894#	3.624	0.248	.067
	Substance × epochs	4, 44	1.329	0.108	.274
	tDCS polarity × substance × epochs	4, 44	8.519	0.436	<.001*

df = degrees of freedom, η ^2^p = partial eta squared, tDCS = transcranial direct current stimulation.

^#^ = Greenhouse−Geisser correction according to violation of sphericity.

**Figure 2.**
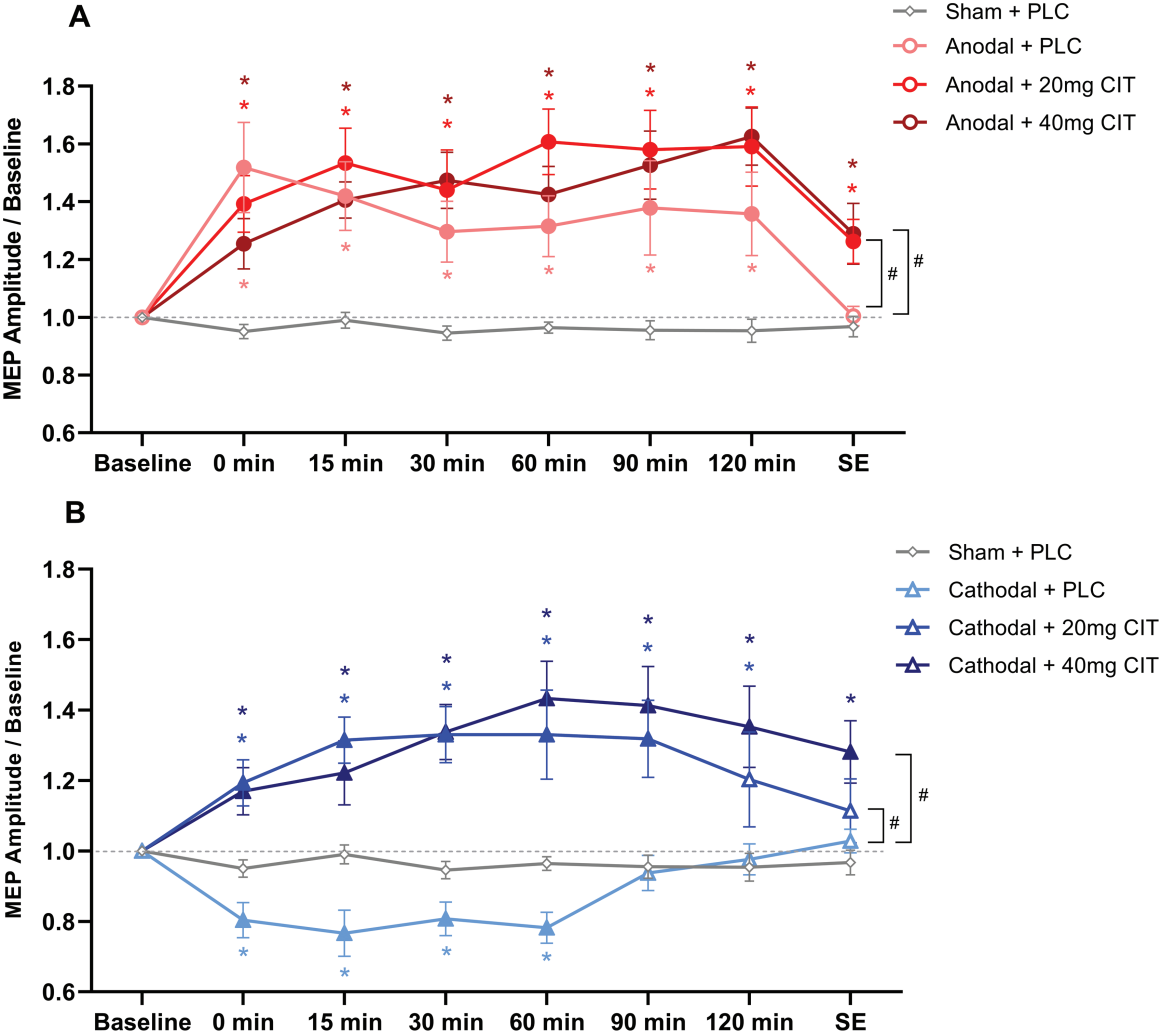
Averaged motor evoked potentials (MEPs) post-stimulation for all intervention conditions and time points. MEPs were obtained before, immediately after, and 15, 30, 60, 90, and 120 minutes after transcranial direct current stimulation (tDCS). In addition, MEPs were recorded at same-day evening (SE), which was 5 to 6 hours after stimulation. (A) For anodal tDCS, compared with the respective baseline values and sham, cortical excitability was increased under placebo medication (PLC) for up to 120 minutes and up to SE under citalopram (CIT), regardless of dosage. Under CIT (20 and 40 mg), the excitability enhancement was larger compared with anodal + PLC only at SE. (B) For cathodal tDCS, compared with the respective baseline values and sham, excitability was decreased under placebo medication for up to 60 minutes. In contrast, under CIT, the excitability-diminishing effect was converted into excitation, and this effect was significant for up to 120 minutes under 20 mg and up to SE under 40 mg CIT. Sham tDCS did not change cortical excitability across time. Error bars represent standard error of the mean. Filled symbols represent a significant difference of MEP amplitudes compared with the respective baselines. Floating symbols represent significant differences between active and sham stimulation conditions (*) and between active conditions (#) (paired *t* test, 2-tailed, *P* < .05).

An exploratory post-hoc *t* test showed that under PLC, compared with respective baseline and sham stimulation, anodal tDCS enhanced cortical excitability for up to 120 minutes. In addition, the excitability-enhancing effect of anodal tDCS was prolonged until SE after stimulation under 20 and 40 mg CIT compared with baseline and sham tDCS. For cathodal tDCS, under PLC medication, excitability was decreased for up to 60 minutes compared with baseline and sham stimulation. Under both CIT conditions, the SSRI converted the excitability-diminishing effect of cathodal tDCS into facilitation. For 20 mg CIT, facilitation was extended until 90 minutes compared with baseline, and until 120 minutes after stimulation compared with sham. Under 40 mg CIT, compared with baseline and sham, the effect was prolonged further until SE (Figure 2).

Regarding dose-dependent effects of CIT, post-hoc comparisons showed a difference between anodal+PLC and anodal+CIT20mg with respect to increased excitability for stimulation combined with CIT at SE (*P* = .015). Likewise, the comparison between anodal+PLC and anodal+CIT40mg demonstrated enhanced excitability at SE under CIT (*P* = .042). However, CIT 20- and 40-mg conditions did not differ for any time point (*P* > .05). In addition, post-hoc *t* tests revealed a difference between cathodal+PLC and cathodal+CIT20mg, showing an increase of cortical excitability for up to 120 minutes under CIT. For cathodal+PLC vs cathodal+CIT40mg, excitability was increased up to SE under CIT. As for the anodal tDCS condition, no difference was found between 20 and 40 mg for cathodal tDCS at any time point (*P* > .05). For all analyses, sham tDCS+PLC did not result in any significant changes of excitability across all time points (*P* > .05).

### Effect of CIT on tDCS-Induced Neuroplasticity (Epoched Data)

The repeated-measures ANOVA showed significant main effects for condition (F_(6,66)_ = 8.559, η _p_^2^ = 0.438, *P* < .001) and epoch (F_(2,22)_ = 10.082, η _p_^2^ = 0.478, *P* = .001) and a significant condition × epoch interaction (F_(12,132)_ = 4.424, η _p_^2^ = 0.478, *P* < .001). Moreover, the ANOVA conducted to specifically explore the impact of CIT on epochs revealed significant main effects for tDCS polarity (F_(1,11)_ = 8.876, η _p_^2^ = 0.447, *P* = .013) and epoch (F_(2,22)_ = 10.810, η _p_^2^ = 0.496, *P* = .001), significant tDCS polarity × substance (F_(2,22)_ = 8.075, η _p_^2^ = 0.423, *P* = .002), and tDCS polarity × substance × epoch interactions (F_(4,44)_ = 8.519, η _p_^2^ = 0.436, *P* < .001). However, no significant effects were found for substance (F_(2,22)_ = 2.193, η _p_^2^ = 0.166, *P* = .135), tDCS polarity × epoch (F_[1.350,14.894]_ = 3.624, η _p_^2^ = 0.248, *P* = .067), and substance × epoch (F_(4,44)_ = 1.329, η _p_^2^ = 0.108, *P* = .274).

For anodal tDCS compared with sham, exploratory post-hoc *t* tests revealed that under PLC, cortical excitability was enhanced significantly for the early (*P* = .004) and late (*P* = .030) epochs. Under both 20- and 40-mg effects, excitability was increased in all epochs compared with sham tDCS. In detail, for 20 mg, excitability was enhanced in the early (*P* < .001), late (*P* < .001), and very late epochs (*P* = .006). For 40-mg CIT, the same pattern of results was observed for the early (*P* < .001), late (*P* < .001), and very late epochs (*P* = .017). For cathodal tDCS compared with sham, a decrease of excitability was observed only for the early epoch under PLC (*P* = .015). For the CIT effect, the excitability-diminishing after-effects of cathodal tDCS were converted into facilitation for the early (*P* < .001) and late epochs (*P* = .006) when 20 mg was administrated and for all epochs when 40 mg was applied (early: *P* = .001; late: *P* = .001; very late: *P* = .005).

**Figure 3.**
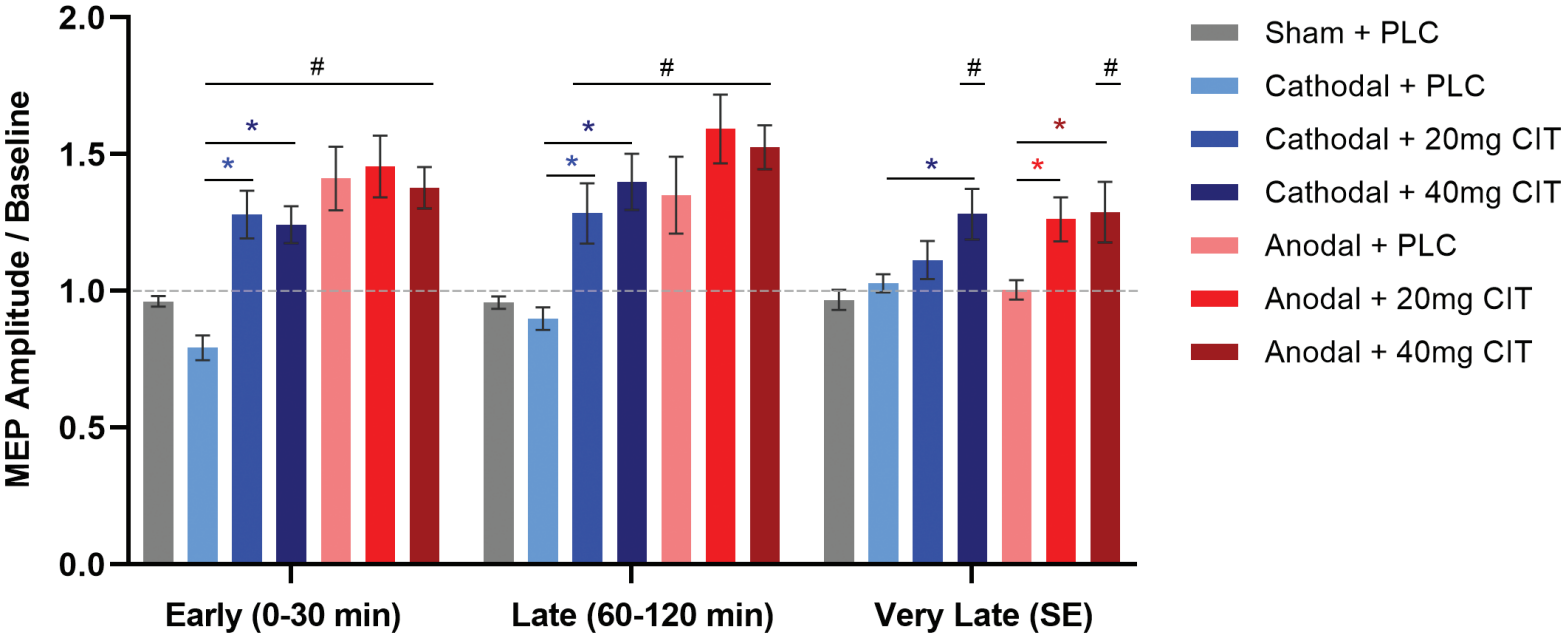
Averaged motor evoked potentials (MEPs) post-stimulation for all intervention conditions and pooled time points (epochs). For anodal transcranial direct current stimulation (tDCS), exploratory post-hoc comparisons indicate an excitability enhancement for the early and late epochs under placebo (PLC) medication compared with sham stimulation. Under citalopram (CIT - 20 and 40 mg), compared with sham stimulation, excitability was increased in all epochs, and compared with anodal + PLC, CIT combined with anodal tDCS resulted in larger excitability enhancements in the very late epoch regardless of CIT dosage. In contrast, for cathodal tDCS, an excitability decrease is observed only for the early epoch under PLC medication compared with sham tDCS. Under 20 mg CIT, the after-effects were converted into facilitation up to the late epoch compared with sham and cathodal + PLC conditions. Under 40 mg CIT, compared with sham and cathodal + PLC, the after-effects were converted into facilitation for all epochs. Error bars represent standard error of the mean. Floating symbols indicate significant differences between active stimulation and sham (#) and between active conditions (*) (paired *t* test, 2-tailed, *P* < .05).

With regard to the dose-dependency effects of CIT, post-hoc showed a difference between anodal+PLC and anodal+CIT20mg (*P* = .015) and, anodal+PLC and anodal+CIT40mg (*P* = .042) for the very late epoch, showing increased excitability for the conditions combined with the substance. However, no difference was identified between 20 and 40 mg for any epoch (*P* > .05). In addition, post-hoc *t* tests revealed a difference between cathodal+PLC and cathodal+CIT20mg, showing increased cortical excitability for early (*P* < .001) and late epochs (*P* = .006) in the CIT condition. For cathodal+PLC vs cathodal+CIT40mg, excitability was increased for early (*P* < .001), late (*P* < .001), and very late (*P* < .015) epochs under CIT. Again, as for anodal tDCS, no difference was found between 20- and 40-mg CIT conditions for cathodal tDCS in any epoch (*P* > .05). For all analyses, sham tDCS+PLC did not result in significant changes (*P* > .05) of excitability across epochs (Figure 3).

## Discussion

In this study, we investigated the dosage-dependent effects of acute 5-HT enhancement on motor cortex plasticity in healthy individuals. The results show that acute augmentation of 5-HT levels by CIT increased and prolonged the LTP-like plasticity induced by anodal tDCS under both 20-mg and 40-mg dosages. In contrast, the LTD-like plasticity induced by cathodal tDCS was switched to LTP-like plasticity, and these after-effects lasted up to the evening of the intervention day only under the 40-mg dosage, showing a dosage-dependent effect for cathodal tDCS.

In healthy humans, the effect of enhancement of 5-HT levels alone (Robol et al., 2004) or in combination with tDCS (Nitsche et al., 2009; Kuo et al., 2016) on cortical excitability was previously investigated. Prior studies assessed the effects of 20 mg CIT in combination with 1 mA tDCS on motor cortical excitability. Acute and chronic application of CIT with that dosage enhanced and prolonged LTP-like plasticity induced by anodal tDCS and converted LTD-like plasticity induced by cathodal tDCS into facilitation (Nitsche et al., 2009; Kuo et al., 2016). The results of the present study not only replicate these effects but show that within the dosage range explored, these effects are largely similar for lower and higher dosages. Besides, other studies with healthy participants demonstrated similar effects of SSRIs in enhancing visual LTP-like plasticity (Normann et al., 2007), memory performance (Prehn et al., 2017), sensorimotor activation (Loubinoux et al., 1999), and motor learning (Loubinoux et al., 2002a, 2002b, 2005), supporting the excitatory and cognition-improving effect of 5-HT.

So far, 7 different families of 5-HT receptors (5-HT_1–7_) have been described in the literature and with the exception of the 5-HT_3_ receptor (ligand-gated ion channel), all others are distinct G protein-coupled receptors. Given this receptor subtype diversity, it is understandable that under physiological and pathological conditions the specific involvement of each receptor may differ (Barnes and Sharp, 1999), and the exploration of their contribution to cortical excitability and plasticity remains a scientific challenge. Nevertheless, the functionality of different 5-HT receptors may help to interpret our findings. Animal in vivo and in vitro studies have revealed that administration of SSRI and 5-HT agonists increase LTP (Mori et al., 2001; Ohashi et al., 2002; Dale et al., 2014), and, in contrast, LTD induction is inhibited or converted into LTP (Kemp and Manahan-Vaughan, 2005), which is in accordance with our findings. Further, a general excitatory effect of 5-HT on the motor cortex has been suggested by genetic or pharmacological manipulation of 5-HT_1A_ receptors (Scullion et al., 2013). This excitatory effect could be partially linked to reduced GABAergic interneuron activity accomplished by 5-HT_1A_ receptors (Llado-Pelfort et al., 2012), which modulate the excitation-inhibition balance in the direction of excitation and thus promote LTP but reduce LTD. Another candidate mechanism of action is LTP promotion by 5-HT_4_ receptor activation. By increasing calcium influx through NMDARs, it has been shown that protein-kinase A (PKA) regulates synaptic plasticity induction (Lau et al., 2009). The amount of PKA-mediated phosphorylation increases with neuronal depolarization, which results in the strengthening of synaptic connections (Schafe et al., 1999), and this mechanism is dynamically regulated depending on 5-HT_4_ receptor activity, as it can either increase or decrease GABA_A_ current with a low or high basal PKA level, respectively (Cai et al., 2002; Ciranna, 2006).

In accordance, 5-HT_4_ receptor agonists have been shown to convert LTD to LTP and also prevent depotentiation of LTP (Kemp and Manahan-Vaughan, 2005), similar to the results we observed for tDCS-induced plasticity. 5-HT_2A_ receptor activation further regulates NMDAR-dependent plasticity (Joo et al., 2015) and increases glutamatergic signaling by augmenting the activity of GluN2A-containing NMDAR (Dantsuji et al., 2019), indicating that a change of calcium dynamics can be achieved also via this mechanism. Another mechanistic explanation for altered tDCS after-effects under CIT is that SSRI reduces membrane potassium conductance via 5-HT_2C_ receptors (Panicker et al., 1991), which enhances the amplitude of depolarizing synaptic responses (Gu, 2002) and leads to enhanced LTP-like plasticity (in the case of anodal tDCS by an increase of calcium influx). In the case of cathodal tDCS, low calcium influx is needed to induce LTD-like plasticity. A respective increase of calcium influx induced by 5-HT_2C_ receptor activation might bring calcium concentration from the LTD- to LTP-inducing range and thus convert the direction of plasticity (Batsikadze et al., 2013b; Mosayebi Samani et al., 2019; Mosayebi-Samani et al., 2020). Considering that tDCS effects are NMDAR- and calcium-dependent (Nitsche et al., 2004; Mosayebi-Samani et al., 2020), these GABA- and NMDAR-dependent mechanisms fit well with the known mechanisms of plasticity induction by tDCS.

To our knowledge, this is the first study to investigate dosage-dependent effects of serotonergic enhancement on tDCS-induced plasticity in healthy humans. In our study, we found a minor dosage-dependent effect only for cathodal tDCS. For anodal tDCS, we did not observe a further enhancement of LTP-like plasticity induced by tDCS compared with 20 mg, probably showing that with the dosages used, we were in a dosage window of relatively homogeneous effects. This does not exclude non-linear effects, including bell-shaped effects, with larger dosages, which are, however, beyond therapeutic dosages and also difficult to achieve in humans because of side effects. Late and longer-lasting facilitatory effects of higher dosages of 5-HT was demonstrated in slices (Huang and Kandel, 2007), and enhanced LTP induction was also observed by higher dosages of a 5-HT_1A/7_ agonist in vivo (Klancnik et al., 1989; Orban et al., 2013). Likewise, a bell-shaped modulation of 5-HT_1A_ receptor agonists on neuronal excitability was shown in vivo; here, the facilitatory effect was reduced under high dosages (Llado-Pelfort et al., 2012). Additionally, a behavioral study in animals showed improved memory consolidation when a medium-dosed 5-HT_7_ agonist was applied, while both low and high dosages had no effect on performance (Meneses et al., 2015). Such non-linear effects were also observed in healthy humans, where 20 mg and 60 mg of paroxetine were administrated to investigate the effects of 5-HT on primary sensorimotor cortex activation using fMRI. The optimal dosage for sensorimotor activation was 20 mg, suggesting an inverted-U dose–response curve (Loubinoux et al., 2002b). These heterogeneous results, compared with the results of the present study, may be due to the specific SSRI applied, since paroxetine acts also on cholinergic and noradrenergic systems, while CIT is a more selective SSRI (Perna et al., 2001). Also, the higher dosage applied in the present study might not have been sufficient to observe non-linearities.

Both SSRIs and tDCS have been proposed to have improving effects on cognition and behavior. SSRIs are well-known pharmacological interventions for treatment of neurological and psychiatric disorders (von Wolff et al., 2013), and their neurogenesis- and plasticity-enhancing effects are related to the improvement of clinical symptoms (Kraus et al., 2017). In addition, there is a growing body of evidence for therapeutic tDCS-induced plasticity for treatment of cognitive impairment (Meinzer et al., 2015; Antonenko and Floel, 2016; Im et al., 2019), and neurological and psychiatric disorders (Meron et al., 2015; Sampaio-Junior et al., 2018; Lindenmayer et al., 2019). Serotonergic enhancement and tDCS are used as treatment approaches in various diseases to reduce symptoms and enhance rehabilitation, and considering that plasticity is impaired in patients with depression (Normann et al., 2007; Grimm et al., 2008; Noda et al., 2018), one important mechanistic foundation is reinstalment/enhancement of plasticity.

Due to synergistic effects of 5-HT and tDCS on plasticity, the option to boost plasticity and functional outcomes by combination of SSRI and tDCS gained increasing attention in the past years. In patients with major depressive disorder, SSRI (50 mg sertraline) combined with anodal tDCS over the dorsolateral prefrontal cortex was superior compared with SSRI or tDCS alone (Brunoni et al., 2013; Padberg et al., 2017). This synergistic effect might not be limited to the treatment of depression. In a case report, bilateral prefrontal cortex tDCS combined with sertraline in obsessive compulsive disorder was efficient for improvement of anxiety and obsessive compulsive symptoms (Palm et al., 2017). Furthermore, in healthy young and older participants, CIT (20 mg) combined with anodal tDCS applied over the right temporoparietal cortex improved immediate memory performance (Prehn et al., 2017). These results are preliminary but promising concerning combined brain stimulation and pharmacological approaches for clinical application. In connection, our findings suggest that CIT up to 40 mg, as applied in clinical practice, combined with tDCS results in LTP-like plasticity enhancement. Our findings encourage future studies to investigate the role of 5-HT augmentation combined with tDCS also for a broader range of symptoms, which might profit from plasticity enhancement, including cognitive and motor rehabilitation.

Some limiting aspects need to be considered for interpreting the results of the present study. Our sample comprised young healthy adults, and thus, these results cannot be completely extrapolated to clinical populations and older age groups. In addition, we did not adjust the medication dosage to the body weight or body mass index, which may potentially increase variability and blur dosage-dependent differences. Although even in clinical practice dosages are typically standardized, it might be advantageous to account for body composition as well as substance serum concentration in future research. Single-dose application might additionally face limitations regarding the investigation of the effects of serotonergic enhancement on plasticity. Interestingly, however, the available evidence for acute and chronic application of CIT (20 mg) combined with tDCS shows quite similar results: CIT increased and prolonged facilitatory plasticity induced by anodal tDCS and converted inhibitory plasticity induced by cathodal tDCS into facilitation in both applications. The main difference between acute and chronic substance administration on cortical excitability was the duration of the effects (Nitsche et al., 2009; Kuo et al., 2016). Moreover, the assessment of specific receptor subtypes as 5-HT_1A_, linked to the antidepressant effects of SSRIs (Lucas et al., 2007), and 5-HT_4_, associated with learning and memory (Hagena and Manahan-Vaughan, 2017; Murphy et al., 2019), might provide more specific mechanistic information in future studies. Finally, the combination of physiological measures and functional effects such as motor learning may help transfer research results from healthy individuals to patients and should be explored in detail by future studies.

This study confirms that acute 5-HT enhancement affects neuroplasticity in healthy humans. CIT enhanced LTP-like plasticity induced by anodal tDCS largely independently from the administered dosage. In contrast, a partially dosage-dependent effect was found for the impact of CIT on cathodal tDCS-induced LTD-like plasticity. Here, CIT converted LTD- into LTP-like plasticity under both dosages, but the effect lasted for several hours only under the higher 40-mg dosage.

## Supplementary Material

pyab035_suppl_Supplementary_Tables
