# Supplementary material for: Dosage-Dependent Impact of Acute Serotonin Enhancement on Transcranial Direct Current Stimulation Effects
Source: Int J Neuropsychopharmacol. 2021 Jun 9;24(10):787–97. doi: 10.1093/ijnp/pyab035 (PMC8538892; doi:10.1093/ijnp/pyab035)
Supplement: pyab035_suppl_Supplementary_Tables [file pyab035_suppl_supplementary_tables.docx]

**Table S1.** Intensity ratings of the tDCS adverse effects per stimulation protocol. Data are presented as mean ± SD.

|  | **Side effects** | **Sham tDCS** | **Anodal tDCS** | **Cathodal tDCS** |
| --- | --- | --- | --- | --- |
| During stimulation | Visual phenomena | 0±0 | 0.05±0.32 | 0±0 |
|  | Itching | 0.75±1.08 | 1.58±1.42 | 1.38±1.03 |
|  | Tingling | 1.0±1.08 | 1.13±0.91 | 0.91±0.82 |
|  | Burning | 0.83±0.98 | 1.13±1.00 | 1.05±1.02 |
|  | Pain | 0.08±0.27 | 0.22±0.53 | 0.19±0.51 |
| Immediately after stimulation | Skin redness | 0.08±0.27 | 0.30±0.51 | 0.25±0.43 |
| 24 hours after stimulation | Skin redness | 0±0 | 0±0 | 0±0 |
|  | Headache | 0±0 | 0±0 | 0±0 |
|  | Fatigue | 0±0 | 0±0 | 0±0 |
|  | Difficulty in concentration | 0±0 | 0±0 | 0±0 |
|  | Nervousness | 0±0 | 0±0 | 0±0 |
|  | Sleep problems | 0±0 | 0±0 | 0±0 |
|  | Others | 0±0 | 0±0 | 0±0 |

**Table S2**. One-way repeated-measures ANOVAs for tDCS adverse effects.

|  | **Factor** | **d.f., error** | **F value** | **η^2^_p_** | ***p* value** |
| --- | --- | --- | --- | --- | --- |
| During stimulation | Visual phenomena | - | - | - | - |
|  | Itching | 1.213, 13.341^#^ | 4.385 | 0.285 | 0.050 |
|  | Tingling | 2, 22 | 0.442 | 0.039 | 0.442 |
|  | Burning | 2, 22 | 0.616 | 0.053 | 0.549 |
|  | Pain | 1.139, 12.527^#^ | 0.579 | 0.050 | 0.482 |
| Immediately after stimulation | Skin redness | 1.135, 14.791^#^ | 1.453 | 0.117 | 0.255 |

df = degrees of freedom, η^2^p = partial eta squared.

^#^Greenhouse−Geisser correction according to violation of sphericity.

**Table S3**. Medication side effects per dosage. Data are presented as mean ± SD.

| **Side effects** | **20 mg**  **Citalopram** | **40 mg**  **Citalopram** | **Placebo** |
| --- | --- | --- | --- |
| Sleepiness | 0.79±1.93 | 1.70±2.36 | 0±0 |
| Dizziness | 0.08±0.40 | 0.66±1.46 | 0±0 |
| Fatigue | 0.29±0.75 | 0.75±1.29 | 0±0 |
| Headache | 0.16±0.48 | 0.25±0.67 | 0±0 |
| Nausea | 0.25±0.89 | 1.16±2.14 | 0±0 |
| Gastrointestinal discomfort | 0.08±0.40 | 0.58±1.41 | 0±0 |
| Metallic taste | 0.12±0.61 | 0.29±0.99 | 0±0 |

**Table S4**. One-way repeated-measures ANOVAs for citalopram side effects per dosage.

| **Factor** | **d.f., error** | **F value** | **η^2^_p_** | ***p* value** |
| --- | --- | --- | --- | --- |
| Sleepiness | 1, 23 | 2.369 | 0.093 | 0.137 |
| Dizziness | 1, 23 | 4.285 | 0.157 | 0.050 |
| Fatigue | 1, 23 | 2.072 | 0.083 | 0.163 |
| Headache | 1,23 | 0.215 | 0.009 | 0.647 |
| Nausea | 1,23 | 3.365 | 0.128 | 0.080 |
| Gastrointestinal discomfort | 1,23 | 3.136 | 0.120 | 0.090 |
| Metallic taste | 1,23 | 1 | 0.420 | 0.328 |

df = degrees of freedom, η^2^p = partial eta squared.

**Table S5.** Blinding assessment data per condition. Participants were asked only if they received real or sham stimulation, not about polarity of stimulation, and not about medication (Chi square test, χ^2^ *=* 4.015, df = 6, *p* = 0.675).

|  | **Sham** | **Cathodal +Placebo** | **Cathodal +20mg** | **Cathodal +40mg** | **Anodal +Placebo** | **Anodal +20mg** | **Anodal +40mg** |
| --- | --- | --- | --- | --- | --- | --- | --- |
| Wrongly guessed | 9 | 4 | 2 | 1 | 2 | 1 | 3 |
| Correctly guessed | 3 | 8 | 10 | 11 | 10 | 11 | 9 |
| Total | 12 | 12 | 12 | 12 | 12 | 12 | 12 |
